# GNMT and Its Regulatory MicroRNAs as Biomarkers and Therapeutic Targets for Metabolic Dysfunction-Associated Fatty Liver Disease and Hepatocellular Carcinoma

**DOI:** 10.3390/ijms27052090

**Published:** 2026-02-24

**Authors:** Yung-Chi Lin, Wei-You Li, Yi-Ming Arthur Chen

**Affiliations:** 1 Graduate Institute of Biomedical and Pharmaceutical Science, College of Medicine, Fu Jen Catholic University, New Taipei City 242, Taiwan; gino.lin@mail.utoronto.ca (Y.-C.L.); laoinwin@hotmail.com (W.-Y.L.); 2 Molecular Medicine, The Hospital for Sick Children, 555 University Avenue, Toronto, ON M5G 1X8, Canada

**Keywords:** GNMT, MASLD, HCC, miR-873-5p, miR-224, therapeutic target, biomarker

## Abstract

Glycine N-methyltransferase (GNMT), a S-adenosylmethionine (SAM)-dependent methyltransferase, is primarily expressed in the liver and plays a key role in regulating liver metabolism and protecting against liver injury. Several studies have shown that deficiency or downregulation of GNMT is strongly associated with the pathogenesis of hepatocellular carcinoma (HCC), highlighting its critical role as a tumor suppressor. Other studies have shown that GNMT is also strongly correlated with the pathogenesis of metabolic dysfunction-associated fatty liver disease (MAFLD). Although many factors regulate GNMT expression, recent studies have identified microRNAs (miRNAs), such as miR-873-5p and miR-224, as key post-transcriptional regulators that directly target GNMT mRNA and suppress its expression in HCC and MAFLD. This review provides an overview of GNMT’s role in liver physiology and how its dysregulation contributes to the progression of HCC and MAFLD, with a focus on the regulation of GNMT by miR-873-5p and miR-224. We also highlight the potential of these two miRNAs as biomarkers and therapeutic targets for HCC and MAFLD, discussing emerging strategies such as antisense-based inhibition, gene therapy, and small-molecule inducers aimed at restoring GNMT expression.

## 1. Introduction

Hepatocellular carcinoma (HCC) and metabolic dysfunction-associated steatotic liver disease (MASLD) are two of the most significant contributors to global liver disease burden. Liver cancer is the sixth most common cancer and is also ranked the sixth in global death rate caused by cancer [1]. HCC is a subtype of liver cancer that originates from hepatocytes and makes up 75% to 85% of all liver cancer cases [2,3], and therefore the main focus for liver cancer therapeutic development. On the other hand, MASLD is the most common chronic liver disease, affecting around 32.4% of the population worldwide [4]. It is characterized by liver steatosis with either one of the metabolic conditions—obesity, type II diabetes mellitus (T2DM), or at least 2 of 7 metabolic risk abnormalities in lean individuals [5]. MASLD has the potential to progress from simple steatosis to steatohepatitis, fibrosis, cirrhosis, and ultimately HCC [6]. It is therefore clinically significant and requires effective treatment. Even though there are increasing numbers of therapeutic strategies such as immune checkpoint inhibitors for HCC and new drug such as resmetirom for metabolic dysfunction-associated steatohepatitis (MASH), the progressive inflammatory form of MASLD, these treatments are generally condition-specific and target either metabolic or immune pathways [7,8].

**Figure 1:**
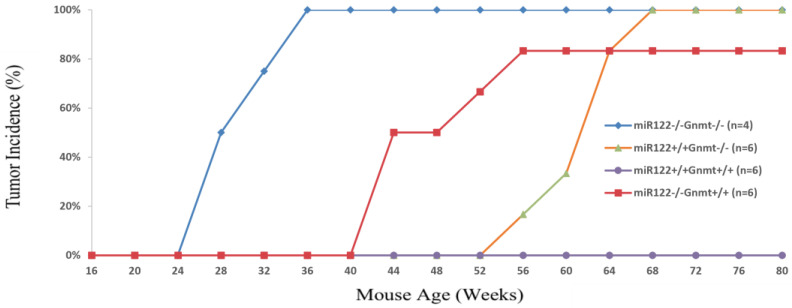
**Tumor incidence in wild type, miR-122 knockout, GNMT knockout, and miR-122/GNMT double knockout mice.** Mice were monitored by ultrasound at 4-week intervals to assess the presence of tumors in liver. For each mouse, the time point at which a tumor was first detected was used to calculate the tumor incidence percentage. The *y*-axis represents the percentage of mice in each group with detectable tumors (n = 4–6 per group).

In contrast, microRNAs (miRNAs) are RNAs that regulate disease-associated genes post-transcriptionally and are becoming more common as therapeutic targets [9]. miRNAs have a dualistic nature in liver diseases, as they could be either oncogenic or tumor-suppressing. For example, miR-122 acts as a tumor suppressor in liver through mechanisms such as targeting IGF-1R, CCNG1, and AKT3, but it also plays a unique role in enhancing HCV replication, which is a major risk factor for HCC [10,11]. GNMT (glycine N-methyltransferase) is a methyltransferase that regulates S-adenosylmethionine (SAM) homeostasis and methylation capacity in the liver [12]. Several studies showed that it also behaves as a tumor-suppressor, exhibiting a cooperative but different role in tumor suppression from miR-122 [13,14]. Double knockout of GNMT and miR-122 in mice leads to accelerated liver tumor formation (Figure 1) [15]. Previously, it has been reported that down-regulation of GNMT contributes to the progression of MASLD and HCC [13,14,16]. On the other hand, both miR-873-5p and miR-224 function have been reported to be able to target at GNMT mRNA [17,18]. Therefore, targeting those miRNAs mentioned above to restore GNMT level presents a unique opportunity to address shared molecular mechanisms in both MSFLD and HCC.

In the following sections, we first summarize how GNMT dysregulation contributes to metabolic and malignant liver disease and briefly note extrahepatic contexts in which GNMT may also be implicated. We then focus on two GNMT-targeting microRNAs, miR-873-5p and miR-224, explicitly distinguishing mechanistic evidence (e.g., reporter assays and gain/loss-of-function studies) from association-based observations in patient cohorts. Finally, we discuss therapeutic strategies to restore GNMT activity and outline a GNMT–miRNA regulatory framework to guide biomarker development and intervention design.

## 2. GNMT Dysregulation (Deficiency) in Different Diseases

GNMT is a highly abundant methyltransferase that uses SAM to methylate glycine to sarcosine, which buffers the cellular SAM pool and links methionine metabolism to global methylation capacity. As a result, inherited loss-of-function variants or acquired downregulation of GNMT can perturb one-carbon metabolism, epigenetic regulation, redox balance, and stress/inflammatory pathways, which together influence susceptibility to liver injury and carcinogenesis.

### 2.1. Congenital GNMT Deficiency

Congenital GNMT deficiency is an autosomal recessive inborn error characterized by continuous hypermethioninemia with plasma SAM being higher than normal, while total homocysteine and S-adenosylhomocysteine typically remain normal or only mildly altered. Since the first clinical descriptions of affected children, only a small number of patients have been reported worldwide, suggesting the rareness or how it might be underdiagnosed [19,20,21].

Clinically, most patients only show phenotypes that are relatively mild, with hepatomegaly and chronically elevated transaminases being the most common. Progressive liver failure is not usually observed, although for some individuals developmental delay and other neurological symptoms have been reported [22,23]. Importantly, several sources note that neurological symptoms are more likely when methionine levels become very high, which provides a pragmatic threshold for closer monitoring and dietary/therapeutic discussion in the context of inherited methylation disorders [21,23]

From a mechanistic perspective, GNMT deficiency reduces the major hepatic ‘sink’ for SAM-dependent methyl transfer, leading to SAM accumulation and altered methylation flux. This biochemical signature can help distinguish GNMT deficiency from other causes of hypermethioninemia like MAT1A deficiency or classical homocystinuria [19,21].

### 2.2. GNMT as a Tumor Suppressor for Hepatocellular Carcinoma

Aside from GNMT’s physiological function in liver as a SAM-dependent methyltransferase, it was also evident that GNMT has a tumor-suppressor role. It was first discovered using mRNA differential display that the GNMT mRNA expression in tumor tissues from patients is downregulated compared to non-tumorous tissues [24]. Northern blot further confirmed that GNMT mRNA was missing or expressed way less in tumor tissues compared to non-tumorous tissues, and monoclonal antibody for GNMT was later developed and verified the downregulation of GNMT in HCC cell line and HCC tumorous tissues by using Western blot and immunohistochemistry [24,25]. Later work developed GNMT-deficient mice (GNMT −/−) model, and GNMT −/− mice developed HCC, further supporting the role of GNMT as a tumor suppressor for HCC [13,14]. Mechanistically, GNMT deficiency leads to aberrant accumulation of SAM, resulting in global DNA hypomethylation and altered expression of oncogenes and tumor suppressor genes [14,26]. GNMT is known to bind to folate and regulate methyl group metabolism, linking it to epigenetic control of gene expression in hepatocytes [27]. In addition, GNMT is known as a 4S PAH-binding protein and plays an important role in the detoxification of environmental toxins as well as food contaminant-aflatoxins and herbal substance- and aristocholic acid type 1 [28,29,30]. Finally, and most importantly, GNMT interacts with several proteins directly and is involved in the oncogenesis pathways. For example, it binds to PREX-2, a Pten inhibitor, and facilitates its degradation through E3 ligase HectH9-mediated proteasomal ubiquitination pathway [31]. In 2017, our group reported that PREX2 protein expression was upregulated in 55% of human HCC samples, while its mRNA level was comparable in tumor and tumor-adjacent tissue, suggesting a post-translational alteration of PREX2 expression. The results reveal a novel mechanism in which GNMT participates in AKT signaling and HCC tumorigenesis by promoting HectH9-mediated PREX2 degradation [31].

### 2.3. GNMT Downregulation Promotes MASLD

Because MAFLD/MASH is a major upstream driver of fibrosis and HCC, clarifying GNMT’s role in metabolic liver injury provides a mechanistic bridge between steatosis and malignant transformation. GNMT also plays a crucial role in maintaining hepatic metabolic homeostasis, and its downregulation has been implicated in the development of metabolic dysfunction-associated steatotic liver disease (MASLD) [32,33]. As a key enzyme in the methionine cycle, GNMT regulates SAM levels and prevents aberrant DNA and protein methylation [34]. GNMT-deficient (GNMT−/−) mice exhibit spontaneous hepatic steatosis, insulin resistance, and progressive liver inflammation [. In addition, genes related to cholesterol uptake (scavenger receptor class B type 1 [SR-B1] and ATP-binding cassette A1 [ABCA1]), intracellular trafficking (NPC1 and NPC2) and excretion (ATP-binding cassette G1 [ABCG1]) were all downregulated in Gnmt (−/−) mice [33]. Mechanistically, excess SAM accumulation disrupts the expression of lipid metabolism regulators such as PPARα and impairs mitochondrial β-oxidation, contributing to lipid accumulation and oxidative stress [34]. Consistent with murine findings, GNMT expression is significantly reduced in liver biopsies from patients with MASLD and non-alcoholic steatohepatitis (NASHs), suggesting a protective role of GNMT against metabolic liver injury and fibrogenesis [35]. It was also found that GNMT localizes to Complex II in the electron transport chain of mitochondria, where it plays a critical role in enhancing mitochondrial respiration and maintaining energy production [35]. By interacting with Complex II, GNMT helps regulate oxidative phosphorylation and prevents mitochondrial dysfunction and oxidative stress, which are key contributors to liver injury and disease progression in MASLD, where GNMT level is downregulated. Another good example of GNMT exerts its influence through protein–protein interaction is Niemann–Pick type C2 protein (NPC2]) [33]. In 2012, Liao et al. using single photon emission computed tomography images of mice injected with I^131^-labeled 6β-iodocholesterol demonstrated that Gnmt (−/−) mice had slower hepatic cholesterol uptake and excretion rates than wild-type mice [33]. Yeast two-hybrid screenings and coimmunoprecipitation assays elucidated that the C conserved region (81–105 amino acids) of NPC2 interacts with the carboxyl-terminal fragment (171–295 amino acids) of GNMT. Confocal microscopy demonstrated that when cells were treated with low-density lipoprotein, NPC2 was released from lysosomes and interacts with GNMT in the cytosol. Overexpression of GNMT doubled the half-lives of both NPC2 isoforms and reduced cholesterol accumulation in cells [33].

Importantly, GNMT loss has also been linked to fibrogenic progression beyond steatosis: in Gnmt (−/−) mice, chronic metabolic injury is accompanied by collagen deposition and induction of profibrotic programs, and normalizing methyl-donor imbalance (e.g., by lowering hepatic SAM) can attenuate both steatosis and fibrosis, supporting a causal role of SAM overload in fibrogenesis [27]. Consistent with this trajectory, reduced GNMT expression has also been reported in advanced chronic liver disease, including cirrhosis, suggesting that GNMT downregulation is not restricted to early MASLD/NASH but may persist (or deepen) with progression toward fibrosis/cirrhosis and cirrhosis-associated HCC risk [36].

Since GNMT was downregulated in the liver tissues from patients suffering with MAFLD, liver fibrosis and cirrhosis, novel therapeutics targeted at inducing GNMT directly or indirectly through downregulating its miRNA should be considered.

### 2.4. Cholangiocarcinoma and GNMT

Beyond hepatocytes, GNMT expression in cholangiocytes raises the question of whether GNMT status also influences bile duct malignancies and clinical outcomes. Although GNMT is most abundant in hepatocytes, it is also detectable in the epithelium of normal bile ducts, indicating that cholangiocytes can express GNMT in vivo. In a study, small resected cholangiocarcinoma cohort assessed GNMT by immunohistochemistry and reported that 6/33 tumors (18.2%) lacked detectable GNMT [37]. More importantly, lower GNMT staining scores were associated with worse survival, and GNMT expression remained a favorable prognostic indicator in multivariable analysis. These findings support the idea that GNMT expression may track with a more differentiated phenotype and/or reduced biological aggressiveness in cholangiocarcinoma, consistent with a protective role of GNMT-linked methyl-group homeostasis in bile duct tumors. However, the cohort size was modest and follow-up studies in a larger cohort is necessary to clarify whether GNMT is a robust prognostic marker.

### 2.5. Pancreatic Cancer and GNMT

Studies showed that there are also high GNMT concentrations in the pancreas, and its activity can be regulated by reduced folate polyglutamates. Its role in pancreas is proposed to be regulating local methyl-group metabolism [38]. In pancreatic ductal adenocarcinoma (PDAC), the most direct evidence points to epigenetic silencing plus reduced expression. In paired human specimens (PDAC vs. matched normal pancreas), it was reported that the GNMT promoter is methylated significantly higher in tumors, as well as reduced GNMT mRNA level in most cases. Moreover, GNMT methylation levels were reported to correlate with tumor grade and disease stage, and demethylation treatment increased the GNMT mRNA level and decreased the PDAC cell viability in vitro [39]. Taken together, these data support the idea that GNMT is regulated epigenetically in PDAC.

Independent transcriptomic analyses are broadly consistent with this directionality. A meta-analysis of multiple pancreatic cancer microarray datasets identified GNMT to be the most downregulated gene consistently and was proposed to be a candidate tumor-suppressing factor in pancreatic cancer [40]. A later study similarly found GNMT to be markedly reduced in most tested pancreatic cancer cell lines and, in early-stage paired patient samples, observed that GNMT expression could drop by ~100-fold or more in a subset of tumors. They also reported that the polyphenol 1,2,3,4,6-penta-O-galloyl-β-D-glucose (PGG) inhibited proliferation and increased GNMT expression in a dose-dependent manner in a PDAC cell line [41]. Together, these studies support a working model in which GNMT repression is recurrent but heterogeneous across PDAC [39,40,41].

### 2.6. Prostate Cancer and GNMT

Similar patterns of altered GNMT abundance have been reported in prostate cancer, underscoring that GNMT’s functional role can be context-dependent. GNMT has been linked to prostate cancer through different studies. In prostate tumor tissues, it was reported that GNMT is abundant in benign prostate (normal/benign prostate hyperplasia) but reduced in a lot of prostate cancer cases, and that the GNMT locus shows loss of heterozygosity (LOH) in a subset of tumors [42]. Furthermore, another study suggested that there are specific GNMT haplotypes associated with prostate cancer risk [43]. Collectively, these studies support GNMT as a candidate node in prostate cancer biology, motivating further research into the mechanism of how GNMT contribute to prostate cancer.

Subsequent studies showed that GNMT is tightly integrated with androgen receptor (AR) biology, a central driver of prostate epithelial growth and prostate cancer progression. An androgen response element (ARE) within the coding region of GNMT’s first exon was mapped, supporting direct AR-responsive transcriptional control [44]. Other studies using AR-positive prostate cancer models provide further evidence for GNMT being regulated by AR [45]. Importantly, AR regulation of GNMT appears to be context-dependent and subject to additional oncogenic constraints. For example, c-MYC overexpression can antagonize aspects of the AR transcriptional program in prostate cancer. The antagonistic relationship between MYC and AR-regulated genes (including GNMT) has been validated in patient samples, highlighting how oncogene activation may reshape GNMT abundance even in AR-driven disease [46].

Beyond AR, PI3K pathway activity adds another layer of regulation that directly intersects with GNMT expression and function. It was reported that GNMT expression is repressed upon PI3K pathway activation, but paradoxically GNMT was required for the onset of invasive prostate cancer in a genetic mouse model, implying that GNMT can support tumor development under specific oncogenic signaling states even when its expression is under negative regulation [47]. Conceptually, this creates a useful framework for a review as follows: GNMT may act as a “signal-integrating” metabolic enzyme in PCa, whose expression and functional requirement vary with AR status, PI3K activity, and broader transcriptional rewiring (e.g., MYC). In this model, “GNMT deficiency” in PCa should be discussed with care—because both reduced expression (e.g., LOH-associated downregulation) and functional dependence (requirement for invasion/onset in certain models) have been observed across different disease contexts and experimental systems [42,47].

## 3. MicroRNAs: The Connection of miRNA to Liver Diseases

### 3.1. miR-873-5p and Liver Diseases

**Figure 2:**
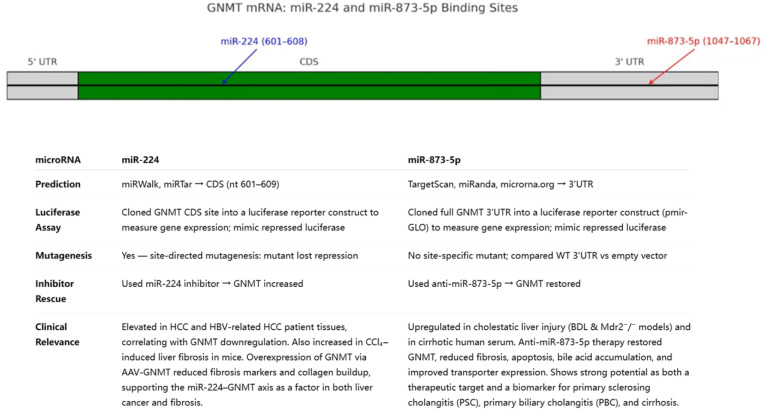
**Summary table for miR-224 and miR-873-5p.** The 2 microRNAs are compared in terms of how they were predicted, how they were proved to target GNMT mRNA, whether inhibiting them would rescue GNMT, and their clinical relevance.

Building on the disease contexts above, we first discuss miR-873-5p, which directly represses GNMT and has been most strongly connected to MASLD/MASH-related metabolic phenotypes. miR-873-5p is a small non-coding RNA located on human chromosome 8p21.3, with a mature sequence of 22 nucleotides. It directly binds to the 3′ untranslated region (3′UTR) of GNMT mRNA to negatively regulate GNMT expression (Figure 2) [18]. Dual-luciferase reporter assays confirmed that transfection with miR-873-5p significantly reduced the luciferase activity of constructs containing the wild-type GNMT 3′UTR binding site, whereas no significant change was observed in constructs with mutated binding sites. This demonstrates that the suppressive effect of miR-873-5p on GNMT is sequence-specific [18].

In MASLD, observational data from patient liver tissues show increased miR-873-5p expression alongside reduced GNMT levels. Mechanistically, functional studies (e.g., GNMT 3′UTR reporter assays and miR-873-5p inhibition in experimental models) support that miR-873-5p directly represses GNMT and can impair mitochondrial function, reduce β-oxidation, and increase oxidative stress [18]. Beyond mitochondrial dysfunction, miR-873-5p also modulates other metabolic pathways, including lipid metabolism, insulin resistance, and inflammation, which contribute to the progression of MASLD [18]. Inhibition of miR-873-5p in mice restores GNMT expression, improves mitochondrial respiration, enhances lipid breakdown, and reduces hepatic fat accumulation and inflammation. These findings suggest that miR-873-5p contributes to MASLD progression by disrupting GNMT-mediated mitochondrial and metabolic homeostasis, and that targeting this microRNA may be beneficial for disease intervention [18,35]. Given its upregulation in NASLD/NASH patient liver tissues, miR-873-5p could also serve as a biomarker for early detection and as an indicator of disease progression. Long-term studies are needed to assess the sustained effects and safety of anti-miR-873-5p treatments, but its potential to improve liver function and reduce fibrosis positions it as a promising therapeutic target in MAFLD. miR-873-5p directly suppresses GNMT, and its effect on mitochondrial function and metabolism may contribute to processes involved in HCC development. This indirect connection between miR-873-5p and HCC via GNMT suggests that further research into this axis could reveal valuable insights into HCC progression and potential therapeutic strategies.

### 3.2. miR-224 and Liver Diseases

In contrast to miR-873-5p (highlighted primarily in metabolic liver disease), miR-224 has been most extensively studied in HCC, including direct GNMT repression and clinical associations. miR-224 is a small non-coding RNA located on chromosome Xq28 (chrX: 151,958,578–151,958,658, minus strand). It directly targets the coding sequence of GNMT mRNA, binding to nucleotides 601–609 (Figure 2). This interaction has been validated by dual-luciferase reporter assays, where miR-224 overexpression significantly reduced luciferase activity in constructs containing the wild-type GNMT sequence, but not in those with mutated binding sites [17].

**Figure 3:**
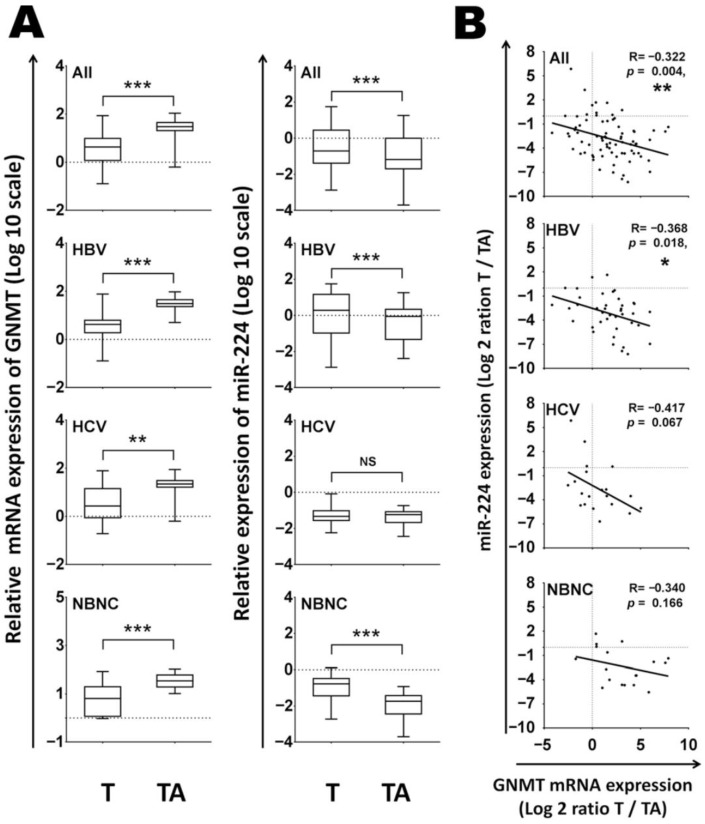
**GNMT mRNA expression was inversely associated with miR-224 levels in HBV-related HCC tissues.** (**A**) The expression levels of GNMT and miR-224 were measured by RT-qPCR in 40 pairs of HBV-associated HCC, 20 pairs of HCV-associated HCC and 18 pairs of HCC patients without hepatitis viral infection. The relative expression fold of GNMT and miR-224 were normalized to TBP and RNU48 RNA, respectively. (**B**) Pearson correlation analysis for miR-224 and GNMT expressions in 40 paired HBV-associated HCC, 20 paired HCV-associated HCC and 18 paired HCC patients without hepatitis viral infection. T, HCC tissue, TA, tumor-adjacent liver tissue. (Modified from [17]). *, **, and *** are indicative of *p* < 0.05, < 0.01, or <0.001, respectively.

In HCC, miR-224 is consistently overexpressed and is associated with advanced tumor stage, increased vascular invasion, and poor patient prognosis. GNMT levels are inversely correlated with miR-224 expression in all HCC tumor patient’s samples, especially in the hepatitis B virus (HBV)-related HCC group (Figure 3). Researchers have found that the detection of miR-224 could be used as an early diagnostic and prognostic biomarker for HCC. Furthermore, overexpression of miR-224 promotes proliferation, migration, and invasion of liver cancer cells [17,32]. Otherwise, GNMT restoration counteracts these effects. Mechanistically, miR-224-mediated GNMT suppression is linked to altered methionine metabolism, reduced SAM levels, and deregulated methylation patterns, contributing to oncogenic transformation. Clinically, high miR-224 expression is associated with HBV-related HCC, suggesting that HBV infection may be linked to its dysregulation [17].

For miR-224, several clinical studies have evaluated diagnostic performance using ROC analysis in patient serum. For example, Lin et al. reported an AUC of 0.880 (sensitivity 86.5%, specificity 76.7%) for serum miR-224 in discriminating early-stage HCC from cirrhosis/CHB/healthy controls [48]. In contrast, for miR-873-5p, the strongest current evidence supports a mechanistic/therapeutic role in MASLD, where inhibition of miR-873-5p restores GNMT and improves mitochondrial and metabolic phenotypes in preclinical models. However, MASLD-focused clinical diagnostic performance metrics (ROC/AUC; sensitivity/specificity for staging, predicting progression, or treatment-response stratification) remain insufficiently established [18].

Previously, Peng et al. reported that miR-224 directly targets early growth response 2 (EGR2) and Acyl-CoA synthetase long-chain family member 4 (ACSL4) during adipogenesis [49]. It regulates fatty acid metabolism through ACSL4 at the terminal differentiation of adipocyte [50]. Moreover, Lendvai et al. found that the expression level of miR-224 was elevated both in steatotic chronic hepatitis C patients and non-viral infected steatosis liver patients compared to control tissues [51]. These observations suggest an association between miR-224 dysregulation and steatotic liver disease; however, direct mechanistic studies in MASLD/MASH models are needed to determine whether miR-224 plays a causal role.

## 4. Therapeutic Potential of miRNA Inhibitors Target at GNMT-Related miRNAs

### 4.1. Antisense-Based and Silent Mutant Inhibition of miRNAs

Having summarized miR-873-5p and miR-224 as GNMT-targeting miRNAs, we next discuss therapeutic strategies aimed at restoring GNMT by inhibiting these miRNAs or bypassing their repression. In MAFLD, miR-873-5p is consistently elevated in both patient liver tissues/serum, and diet-induced mouse models, accompanied by a reduction in GNMT expression. Functional studies demonstrate that this upregulation disrupts GNMT-mediated mitochondrial function, leading to impaired fatty acid β-oxidation, increased reactive oxygen species production, and hepatic lipid accumulation. Inhibition of miR-873-5p in experimental models restores GNMT levels, improves mitochondrial respiration, reduces oxidative stress, and alleviates steatosis. These findings indicate that miR-873-5p contributes to MASLD pathogenesis through suppression of GNMT, and that targeting this miRNA may offer a strategy to restore metabolic and mitochondrial homeostasis in the liver [48].

In vitro, antisense inhibition of miR-224 in HCC cell lines restored GNMT expression and reduced proliferation, colony formation, migration, and invasion, whereas GNMT knockdown reversed these effects, indicating that the oncogenic actions of miR-224 are largely GNMT-dependent. In vivo, anti–miR-224 oligonucleotide delivery to HBV-related HCC xenograft mouse models significantly reduced tumor growth and improved liver function, with more pronounced benefits in HBV-associated cases, consistent with the strong clinical association between miR-224 elevation and HBV infection. Additional experiments in CCl_4_-induced liver injury models showed that miR-224 induction coincided with GNMT suppression, and that AAV-mediated GNMT expression not only mitigated fibrosis but also downregulated miR-224, suggesting a reciprocal regulatory relationship during liver injury and tumorigenesis [17]. Together, these results demonstrate that antisense oligonucleotides targeting disease-associated miRNAs can restore GNMT expression and counteract the downstream oncogenic and metabolic effects in both cancer and metabolic liver disease contexts. Furthermore, the therapeutic potential of the miR-224 binding site silent mutation in GNMT coding sequences (CDSs) has been evaluated. The researchers used RegRNA 2.0 and miRbase database to analyze the minimal side effects of mutations of GNMT CDS and determined a silent mutation sequence (UCAGAUCUC). Both GNMT mRNA and protein expression analyses demonstrated that the transit transfection of silent mutant type of GNMT cDNA could evade miR-224 repression in HEK293T cell, in contrast to wild-type GNMT cDNA transfection. This pioneering study provides a novel approach for gene therapy [17].

### 4.2. AAV-Mediated GNMT Delivery

Beyond antisense inhibition, GNMT can also be restored more directly via gene delivery approaches. In the study investigating miR-224 and GNMT in HCC, an adeno-associated virus (AAV) vector carrying the human GNMT coding sequence with a C-terminal FLAG tag was constructed and injected into mouse liver. In the HBV-related HCC and CCl_4_-induced liver injury models, AAV-GNMT overexpression significantly reduced tumor growth, restored (SAM) levels, and improved global methylation status in tumor tissue [17].

In the CCl_4_ model, AAV-GNMT administration prior to toxin exposure decreased miR-224 expression, lowered collagen I, α-SMA, and TGF-β1 mRNA levels, and attenuated hepatic fibrosis, as confirmed by Masson’s trichrome staining. Fibrosis severity was milder in the AAV-GNMT group than in the AAV-eGFP or untreated CCl_4_ groups. The data indicate that AAV-mediated GNMT delivery not only counteracts miR-224-driven suppression of GNMT in HCC but also confers protective effects against liver injury and fibrosis [17].

### 4.3. Small-Molecule Inducers of GNMT

In addition to nucleic-acid and gene-delivery approaches, small molecules that induce GNMT expression offer an orthogonal strategy that may be easier to scale and administer. 1,2,3,4,6-Penta-O-galloyl-β-D-glucopyranoside (PGG) is a bioactive compound identified from Paeonia lactiflora Pall, a traditional Chinese medicinal herb. It was discovered through a high-throughput screening system designed to enhance the expression of GNMT, a key enzyme often downregulated in HCC [51]. PGG functions as a potent GNMT inducer, significantly increasing GNMT expression both in vitro and in vivo. This action restores SAM levels and improves cellular methylation status. The compound exhibits notable anti-cancer effects, inducing apoptosis in HCC cells and enhancing the sensitivity of these cells to sorafenib, a chemotherapy drug commonly used in liver cancer treatment [52]. These findings suggest that PGG holds promise as a therapeutic agent for HCC and other metabolic disorders associated with GNMT deficiency.

K117 is a newly developed synthetic small molecule discovered through a GNMT promoter-based high-throughput screening platform as a potent inducer of GNMT expression [50]. In preclinical studies, K117 markedly reduced tumor volume in hepatocellular carcinoma xenograft models and alleviated hepatic steatosis in experimental models of metabolic dysfunction-associated fatty liver disease, indicating therapeutic potential across both malignant and metabolic liver disorders. Mechanistic investigations revealed that K117 functions as a MYC inhibitor, suppressing MYC transcriptional activity and thereby relieving MYC-mediated repression of the GNMT promoter. This leads to robust GNMT upregulation, which dampens oncogenic signaling in HCC cells and improves mitochondrial and metabolic homeostasis in the liver. Overexpression of MYC negated K117’s ability to induce GNMT and abolished its antiproliferative effects, confirming that MYC inhibition is central to its dual antitumor and metabolic benefits [50].

### 4.4. Summary

We propose a GNMT-centered regulatory framework in which disease-associated induction of miR-873-5p and miR-224 converges on suppression of GNMT, shifting hepatocyte metabolic homeostasis toward a state that favors fibrosis progression and malignant transformation. In this framework, chronic metabolic and inflammatory stress in MASLD/MASH is associated with elevated miR-873-5p and reduced GNMT, which can impair one-carbon buffering and mitochondrial metabolic programs, thereby promoting oxidative stress and fibrogenic signaling. As disease advances toward HCC, miR-224-mediated repression of GNMT may further reinforce pro-tumor behavior by sustaining metabolic imbalance and downstream gene-expression programs linked to proliferation and invasion. This framework integrates mechanistic evidence (direct GNMT targeting and functional perturbation) with human association data, and it generates testable predictions: (i) restoring GNMT activity or inhibiting GNMT-targeting miRNAs should improve both metabolic readouts (e.g., methyl-donor balance/mitochondrial function) and disease phenotypes (fibrosis burden and tumor aggressiveness); and (ii) the therapeutic/biomarker utility of each miRNA will be stage- and context-dependent, with miR-873-5p more informative for fibrotic/cholestatic progression and miR-224 more directly informative for HCC-related outcomes.

Nevertheless, prospective cohort validation and standardized pre-analytic/normalization procedures will be essential before either miRNA can be adopted as a standalone clinical biomarker.

Therapeutic strategies to restore GNMT function—whether by blocking inhibitory miRNAs, delivering GNMT via viral vectors, or inducing its expression with small molecules—have shown promise in preclinical settings across MASLD/MASH and HCC. The dual role of GNMT in cancer suppression and metabolic regulation makes it an attractive target for integrated liver disease therapies. Future research should focus on optimizing delivery systems, minimizing off-target effects, and validating these strategies in well-designed clinical studies.

## Data Availability

No new data were created or analyzed in this study. Data sharing is not applicable to this article.
